# Development of Plant-Based Burgers with Partial Replacement of Texturized Soy Protein by *Agaricus bisporus*: Effects on Physicochemical and Sensory Properties

**DOI:** 10.3390/foods13223583

**Published:** 2024-11-09

**Authors:** Géssica Hollweg, Pamela Cristiele Oliveira Trindade, Bibiana Alves dos Santos, Milena Padilha, Priscila Rossato Fracari, Sarita Correa Rosa, Alexandre José Cichoski, Paulo Cezar Bastianello Campagnol

**Affiliations:** Department of Technology and Food Science, Federal University of Santa Maria, Santa Maria 97105-900, RS, Brazil; gessicahollweg@gmail.com (G.H.); pamelactrindade@gmail.com (P.C.O.T.); bialvesantos@gmail.com (B.A.d.S.); mypadilha5@gmail.com (M.P.); priscila_434@hotmail.com (P.R.F.); saritacorrearosars@gmail.com (S.C.R.); cijoale@gmail.com (A.J.C.)

**Keywords:** plant-based alternatives, sensory evaluation, texture analysis, moisture content, cooking yield, consumer acceptance

## Abstract

This study aims to develop plant-based burgers with partial replacement of texturized soy protein (TSP) by *Agaricus bisporus* mushrooms at proportions of 5%, 10%, 15%, and 20%. The substitution was evaluated regarding its impact on the burgers’ chemical composition, texture, color, cooking performance, and sensory properties. Chemical analyses showed a significant increase in moisture content starting from the 10% substitution level, contributing to improved juiciness. Protein content remained similar to the control until the 15% substitution level, while the fat content showed no significant variation among treatments. The texture profile indicated reduced hardness in burgers with mushroom enrichment, particularly at 5% and 10%, leading to a more tender product. Color analysis revealed a reduction in lightness (L*) and red intensity (a*) with increased mushroom levels. Sensory analysis showed that burgers with up to a 15% substitution level maintained consumer acceptance comparable to the control, with attributes such as “softness”, “pleasant color”, and “good appearance” positively correlated with consumer acceptance. The findings indicate that *Agaricus bisporus* mushrooms can be effectively used as a partial substitute for TSP in plant-based burgers, enhancing sensory properties without compromising quality. This substitution offers a promising approach to diversifying ingredients in plant-based products while maintaining desirable characteristics for consumers.

## 1. Introduction

Plant-based alternatives to meat have continued to develop, driven by the growing predisposition of consumers to reduce or avoid the consumption of animal-derived products in their diets [1,2]. This shift is motivated by various factors, including lifestyle choices (vegetarian, vegan, and flexitarian), environmental concerns, animal welfare ethics, economic factors, and potential health benefits [3]. Additionally, plant-based options are increasingly chosen by a broader range of consumers as an option alongside traditional foods. However, despite its popularity, texturized soy protein (TSP) presents some limitations, including the potential for allergenicity, the presence of strong beany flavors that may be undesirable in certain applications, and textural challenges when creating products with meat-like qualities [4]. These drawbacks underscore the potential benefits of partially replacing TSP with *Agaricus bisporus* mushrooms, which could improve flavor, texture, and nutritional profile. According to projections by the Food and Agriculture Organization of the United Nations (FAO), the world population may reach 9 billion people by 2050. In this scenario, it will be crucial to intensify global food production, which will need to increase by approximately 30% to meet the rising global demand [5].

In light of these challenges, plant-based meat alternatives have emerged as a way to meet new consumer demands and contribute to the sustainability of food production [6]. Texturized soy protein (TSP) has been widely used in meat analogs due to its similarity in texture and appearance. TSP is typically produced from defatted soybean flour through extrusion, with conditions often set between 120 and 150 °C and pressures around 20–25 MPa, enabling fibrous, meat-like texture suitable for plant-based applications [4,7]. This process results in a product rich in protein, fiber, and essential minerals that contribute to human nutrition [8]. This versatility makes TSP an excellent choice as a base for plant-based products. However, there is a growing interest in exploring new ingredients that can diversify plant-based products and provide differentiated sensory characteristics, justifying the partial replacement of TSP. In this context, edible mushrooms, particularly *Agaricus bisporus*, have gained relevance in terms of the development of plant-based products due to their rich nutritional profile. *Agaricus bisporus* provides essential vitamins, such as riboflavin (B2) and niacin, and minerals, like selenium, copper, and potassium, which contribute to its antioxidant properties and support immune health. Selenium, in particular, works synergistically with vitamin E to protect cells from oxidative damage, while potassium aids in maintaining healthy blood pressure levels. Additionally, mushrooms are one of the few non-animal sources of vitamin D, especially when exposed to UV light, further enhancing their nutritional value and appeal as an ingredient in plant-based foods [9,10].

The *Agaricus bisporus* mushroom (brown variety), commonly known as Portobello, presents, when at an advanced stage of maturation, a dark brown coloration, an intense flavor and a firm and fibrous texture, characteristics that resemble meat and which make it a promising alternative for plant-based products [11]. This sensory similarity to meat contributes to consumer acceptance and offers a differentiated alternative in terms of flavor and texture.

This study innovates by exploring the use of *Agaricus bisporus* mushrooms as a partial substitute for TSP in plant-based burgers, with the hypothesis that mushroom inclusion will enhance the sensory characteristics of the burgers while maintaining their physicochemical integrity. Thus, the objective of this study was to evaluate the effect of partially replacing TSP with *Agaricus bisporus* mushrooms at levels of 5%, 10%, 15%, and 20%, aiming to assess whether this substitution is feasible in terms of both the physicochemical and sensory properties of the final product.

## 2. Materials and Methods

### 2.1. Materials

Dark texturized soy protein, containing 50% protein, was purchased from Camil Alimentos S.A. (Camaquã, Brazil). The *Agaricus bisporus* mushrooms used in the research were supplied by Tuppan Champignons Sul Agroindustrial Ltd.a. (Tupanciretã, Brazil). Teff flour was obtained from Giroil (Entre-Ijuís, Brazil). Modified cassava starch (AMD 21) and modified corn starch (AMD 11) were purchased from Adicel Indústria e Comércio Ltd.a. (Belo Horizonte, Brazil). The other ingredients were acquired from the local Santa Maria (Brazil) market.

### 2.2. Plant-Based Burger Preparation

For the preparation of the control burger, the following ingredients were used on a wet basis: texturized soy protein (65.3%), vegetable fat (15%), modified cassava starch (AMD 21) (1%), modified corn starch (AMD 11) (1%), carboxymethylcellulose (1%), teff flour (10%), sodium chloride (1.2%), garlic powder (0.2%), onion powder (0.1%), black pepper (0.1%), monosodium glutamate (0.3%), meat flavoring (0.05%), liquid smoke (0.15%), and water (4.6%). In the modified treatments, part of the TSP was replaced by *Agaricus bisporus* mushrooms, which were added at levels of 5% (M_5%_), 10% (M_10%_), 15% (M_15%_), and 20% (M_20%_), corresponding to a proportional reduction of the soy protein.

The TSP used in all treatments was hydrated in water at a ratio of 1:2 (protein). The TSP was added to boiling water, and acetic acid (vinegar) with a 4% acetic acidity concentration was incorporated at 1:3.3 (vinegar: protein). The mixture was kept boiling for 5 min. After this period, the TSP was strained to remove excess water and rinsed quickly under running water to reduce the soy protein’s intense flavor and odor characteristic, as determined through preliminary laboratory trials demonstrating improved sensory acceptance. The *Agaricus bisporus* mushrooms used in the formulations were sanitized by gently wiping them with a moistened paper towel to remove adhered dirt. After cleaning, the stipe and ring were removed, leaving only the hymenium and cap for use. The mushrooms were placed on a rack over water preheated to 90 °C, this was then enclosed to allow exclusive contact with the steam for 4 min. This controlled steaming minimizes contamination while preserving structural integrity, as immersion in sanitizing solutions could compromise the mushrooms’ texture. After this process, the mushrooms were reduced in size using a blade mixer for 1 min to achieve a uniform particle size suitable for the formulation.

All ingredients were placed in a container, where the mixture was homogenized to ensure uniform incorporation of all components. Then, the mixture was covered with plastic wrap and stored at 4 °C for 20 min to facilitate molding. The mixture was molded into patties with a diameter of 11 cm and a thickness of 1.5 cm using a conventional burger molder (HP 112, Picelli, São Paulo, Brazil). The plant-based burgers (100 g each) were individually packed in high-density polyethylene bags (18 × 14 cm; 50 µm thickness; oxygen transmission rate: 1434 cm^3^/m^2^ per day; water vapor transmission rate: 0.6 g/m^2^ per day; Extrusa-Pack, São Paulo, SP, Brazil) and sealed using a sealing machine (Selovac, São Paulo, Brazil). The packaged plant-based burgers (30 per treatment) were placed on polystyrene trays and stored at −18 °C until analysis.

### 2.3. Chemical Composition

The chemical composition of the plant-based burgers was determined in triplicate immediately after production. The fat content was determined using the method described by Bligh and Dyer [12]. The moisture (950.46), ash (920.153), and protein (992.15) contents were quantified using AOAC [13] methods.

### 2.4. Texture Profile Analysis

The texture profile of the plant-based burgers was evaluated using a Stable Micro Systems Texture Analyzer TAX-T2 (Godalming, UK). Based on Bourne’s criteria [14], the parameters analyzed included hardness (N), springiness (dimensionless), cohesiveness (dimensionless), gumminess (N), and chewiness (N). Ten cylindrical samples (approximately 2.5 cm in diameter and 1.0 cm in height) were tested for each treatment. A P/40 probe (40 mm diameter) was used, with samples compressed in two successive cycles to 50% of their original height at a test speed of 1 mm/s.

### 2.5. Instrumental Color Analysis

The color parameters L*, a*, and b* of the plant-based burgers were determined using a colorimeter CR-400 (Konica Minolta Sensing Inc., Osaka, Japan), operating in spectral reflectance mode, with a 10° observation angle, D65 illuminant, and a 1.5 cm circular aperture. Two units were analyzed for each treatment, and five readings were taken at different points on the surface of each sample.

### 2.6. Cooking Yield and Shrinkage Analysis

The cooking yield of the plant-based burgers was measured by weighing them before and after cooking. Five units were thawed at 4 °C for 12 h for each treatment. Cooking was performed on an electric grill with temperature control set to 180 °C. Each side of the burger was cooked for two minutes until reaching an internal temperature of 72 °C. The cooking yield was calculated as follows, according to Berry’s formula [15]:

$$\%\mathrm{Cooking}\;\mathrm{Yield}=\frac{\left(\mathrm{weight}\;\mathrm{after}\;\mathrm{cooking}\right)}{\left(\mathrm{weight}\;\mathrm{before}\;\mathrm{cooking}\right)}\times 100$$

To evaluate the shrinkage percentage, the diameter of five plant-based burgers per treatment was measured before and after cooking. The shrinkage percentage was calculated as follows, using an equation proposed by Berry [15]:

$$\%\;shrinkage=\frac{\left(\mathrm{diameter}\;\mathrm{of}\;\mathrm{raw}\;\mathrm{sample}-\mathrm{diameter}\;\mathrm{of}\;\mathrm{cooked}\;\mathrm{sample}\right)}{\mathrm{diameter}\;\mathrm{of}\;\mathrm{raw}\;\mathrm{sample}}\times 100$$

### 2.7. pH and Water Activity (aW) Analysis

For pH analysis, 5 g of ground sample was transferred to a beaker, to which 50 mL of distilled water was added. The pH of the solution was measured using a pH meter (Model 130 MA; Mettler Toledo, Barueri, Brazil), previously calibrated with buffer solutions of pH 4.0 and pH 7.0 (Merck, Darmstadt, Germany). Water activity (aW) was measured using an AquaLab Series 4 TEV analyzer (Decagon Devices, Inc., Pullman, WA, USA). The pH and aW were determined in triplicate.

### 2.8. Sensory Analysis: Acceptance Test and Check-All-That-Apply (CATA)

The sensory analysis took place in a laboratory specialized for this type of evaluation. Consumers performed the tests in individual booths under white, fluorescent lighting. Samples were cooked using a stainless-steel grill for 2.5 min at 180 °C to ensure complete cooking. Each participant received a sample of approximately 10 g per treatment, which was coded with random three-digit numbers and served monadically [16]. One hundred consumers participated in the analysis, including 32 men and 68 women aged between 18 and 58. All participants signed a sensory analysis informed consent form before the evaluation. During the evaluation of the samples, consumers were instructed to examine each sample through olfactory perception to verify the aroma, as well as to perform a visual analysis and evaluate the flavor [17]. Between tastings for each sample, consumers were asked to cleanse their palate with room temperature water, and saltine crackers were also provided to aid in palate cleansing. Initially, consumers performed an acceptance test, in which they rated the samples using an unstructured hedonic scale, with scores ranging from 1 (dislike extremely) to 9 (like extremely) for the following parameters: color, aroma, flavor, texture, and overall acceptance. Subsequently, consumers participated in the CATA test, selecting descriptors they considered relevant for characterizing the samples from different treatments. The CATA questionnaire contained the following descriptors: mild aroma, greasy appearance, salty taste, soy flavor, soft, rancid aroma, good appearance, pleasant taste, soy aroma, pleasant texture, mild flavor, mushroom aroma, good chewiness, rancid taste, hard, pleasant color, brown color, meat aroma, meat flavor, unpleasant color, mushroom flavor, unpleasant aroma, smoky aroma, unpleasant taste, and smoky flavor.

### 2.9. Statistical Analysis

The experiment was repeated three times (n = 3). A general linear model ANOVA was used to evaluate physicochemical data, with treatments considered as fixed factors and repetitions treated as a random factor. Tukey’s test was applied for comparisons between means, adopting a significance level of 5%. A mixed linear model was used to analyze sensory acceptance test data, with treatments considered fixed factors and consumers considered random factors. Again, Tukey’s test was used for pairwise comparisons, with a significance level of 5%. The representation of CATA data was carried out using correspondence analysis. In contrast, principal coordinates analysis was used to visualize the correlation between CATA descriptors and acceptance ratings, using tetrachoric and biserial correlation. All statistical analyses were performed using XLSTAT 2019.1 software (Addinsoft, Paris, France).

## 3. Results and Discussion

### 3.1. Chemical Composition

In the evaluation of the chemical composition of the burgers (Table 1), a significant increase in moisture content was observed starting from the 10% level of TSP replacement with mushroom, compared with the control. This increase can be attributed to mushrooms containing more than 90% water in their composition [18]. This increase in moisture is desirable, as it contributes to the plant-based burger’s juiciness and, therefore, to better sensory acceptance of the product by consumers [19].

Regarding protein content, only the 20% replacement of TSP with mushroom resulted in a significant reduction. This indicates that, up to the 15% replacement level, the plant-based burgers maintained protein levels similar to the control, which is a positive point for the nutritional quality of the product [19]. The reduction observed in the 20% mushroom treatment is due to the lower protein concentration of the mushroom (1.72%) compared with TSP (50%) [11].

The fat content of the treatments did not show significant differences, ranging from 14.9% to 16.4%, which is due to the constant proportion of vegetable fat (15%) added to the formulations. The other ingredients, such as TSP and mushrooms, have nearly zero fat content [11].

Finally, the ash content decreased with increased TSP replacement with mushrooms. Only the treatment with 20% mushroom showed a significant reduction compared with the control. This is due to the mushroom’s having a lower mineral density than TSP, which contains a higher concentration of minerals due to its dehydration and concentration process [20].

### 3.2. pH and aW

**Figure 1:**
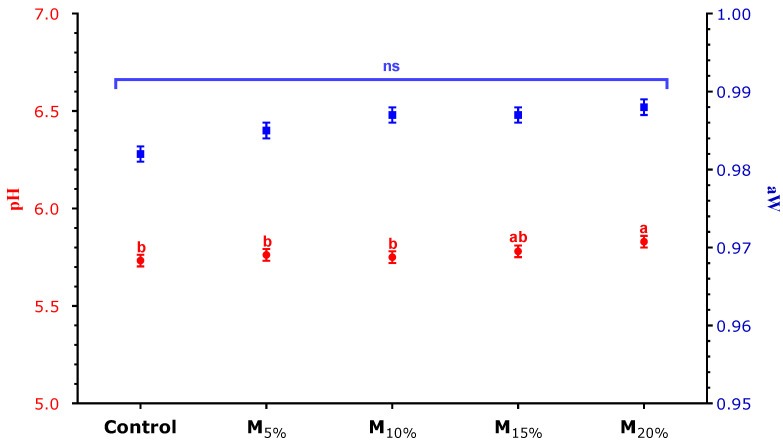
pH (red font) and aW (blue font) of the plant-based burgers. Averages (±standard error of the mean) followed by the same letters did not show any significant difference (*p* > 0.05) by Tukey’s test. Batches: control: 65.3% hydrated texturized soy protein (1:2 protein: water); M_5%_, M_10%_, M_15%_, and M_20%_: 5, 10, 15, and 20% substitution of hydrated texturized soy protein by raw mushroom, respectively. n.s. (not significant).

The data for pH and water activity (aW) of the plant-based burgers are presented in Figure 1. It was observed that pH values increased with the level of TSP replacement with mushroom, with only the M_20%_ treatment showing a significantly higher value (*p* < 0.05) than the control. This increase in pH can be explained by the treatment of TSP with vinegar during the hydration process, which resulted in greater acidity in the control and treatments with lower replacement levels. The obtained pH, ranging from 5.7 to 6.2, can be considered normal for this plant-based product [21].

The aW of the plant-based burgers ranged from 0.98 to 0.99, with no significant difference between treatments. These values reflect the high water content of the mushrooms and the necessary hydration of TSP to obtain the desired texture. The high aW reinforces the importance of storing the products under freezing conditions to control microbial growth [22,23].

### 3.3. Instrumental Color

**Figure 2:**
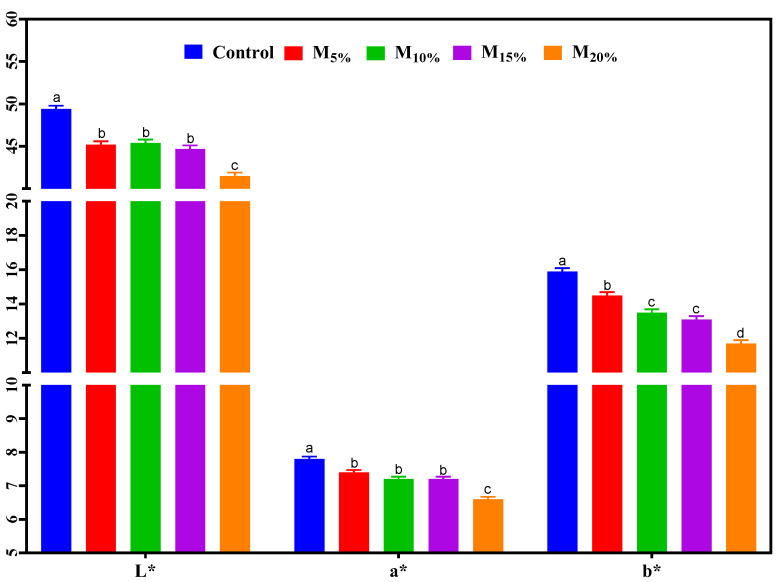
Instrumental color (L*, a* and b*) of the plant-based burgers. Averages (±standard error of the mean) followed by the same letters did not show any significant difference (*p* > 0.05) by Tukey’s test. Batches: control: 65.3% hydrated texturized soy protein (1:2 protein: water); M_5%_, M_10%_, M_15%_, and M_20%_: 5, 10, 15, and 20% substitution of hydrated texturized soy protein by raw mushroom, respectively.

**Figure 3:**
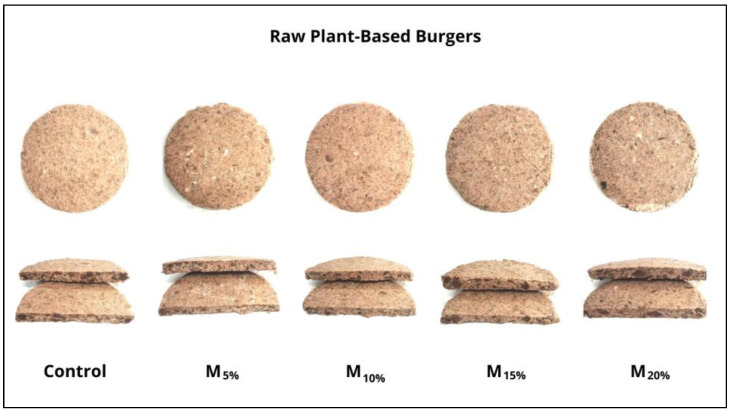
External and internal appearance of raw plant-based burgers. Batches: control: 65.3% hydrated texturized soy protein (1:2 protein: water); M_5%_, M_10%_, M_15%_, and M_20%_: 5, 10, 15, and 20% substitution of hydrated texturized soy protein by raw mushroom, respectively.

The results of the instrumental color analysis are presented in Figure 2. All treatments showed significant differences in color parameters. The L* values (lightness) decreased consistently with increased TSP replacement with mushrooms. The control showed higher lightness, while the M20% treatment had the lowest value. This reduction in lightness is associated with the natural color of the mushrooms, which is darker, while TSP, due to its water retention capacity, contributes to a brighter appearance. The a* values (redness) decreased with higher mushroom content, reflecting a reduction in the red hue intensity. The control exhibited the highest red intensity, attributed to the caramel coloring present in TSP, whereas including the brownish-gray mushroom decreased the a* values. Similarly, b* values (yellowness) also declined as mushroom levels increased, resulting in burgers with a darker tone and less intense red and yellow hues. The external and internal appearance of the raw plant-based burgers can be visualized in Figure 3.

These chromatic changes may affect consumer acceptance, as color is a primary factor influencing the perception of plant-based products [24]. Studies indicate that consumers often associate lighter and redder hues with traditional meat products, which may enhance the initial appeal of plant-based alternatives designed to mimic meat [25,26]. However, for consumers who prefer natural and minimally processed products, the darker shades introduced by mushroom substitution can signal authenticity and align with expectations for natural ingredients. This alignment with consumer expectations can enhance the sensory perception and acceptance of plant-based products with natural color variations [27,28].

### 3.4. Texture Profile

**Figure 4:**
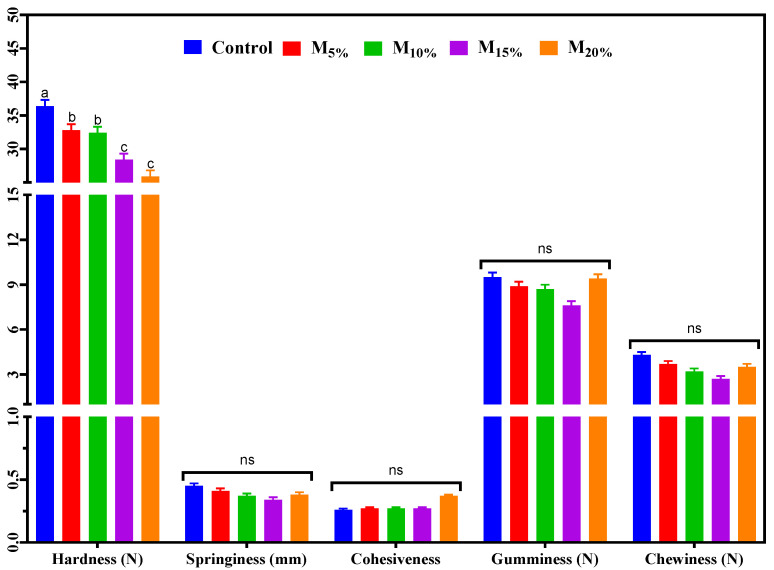
Textural parameters of the plant-based burgers. Averages (±standard error of the mean) followed by the same letters did not show any significant difference (*p* > 0.05) by Tukey’s test. Batches: control: 65.3% hydrated texturized soy protein (1:2 protein: water); M_5%_, M_10%_, M_15%_, and M_20%_: 5, 10, 15, and 20% substitution of hydrated texturized soy protein by raw mushroom, respectively. n.s. (not significant).

The texture parameters of the plant-based burger samples are presented in Figure 4. There were no significant differences between treatments for springiness, cohesiveness, gumminess, and chewiness. However, there was a significant difference in hardness, with the control showing the highest value compared with the other treatments. It was observed that the formulations with mushroom proportions of 5% and 10% (respectively, M_5%_ and M_10%_) had similar hardness values to each other, as did the formulations with proportions of mushroom of 15% and 20% (respectively, M_15%_ and M_20%_). The higher hardness observed in the control can be attributed to the exclusive presence of TSP, which maintains a relatively firm structure even after hydration. As mushrooms replaced TSP, hardness values decreased. This reduction in hardness may be due not only to the inherently softer texture of mushrooms but also to potential structural changes in the matrix that result from the higher water content of mushrooms. This additional moisture could decrease matrix cohesion, leading to a softer texture. Furthermore, interactions between mushroom components and TSP proteins may also contribute to these textural changes, influencing the product’s overall firmness [10,29,30]. These results indicate that replacing TSP with mushrooms contributes to a balanced and more pleasant texture for consumers, facilitating handling and providing good chewiness, which are desirable characteristics for plant-based burgers [22].

### 3.5. Shrinkage and Cooking Yield

**Figure 5:**
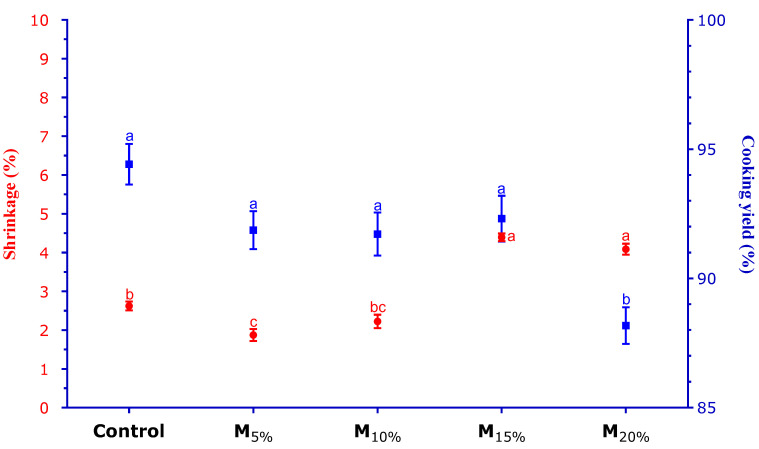
Shrinkage (red font) and cooking yield (blue font) of the plant-based burgers. Averages (±standard error of the mean) followed by the same letters did not show any significant difference (*p* > 0.05) by Tukey’s test. Batches: control: 65.3% hydrated texturized soy protein (1:2 protein: water); M_5%_, M_10%_, M_15%_, and M_20%_: 5, 10, 15, and 20% substitution of hydrated texturized soy protein by raw mushroom, respectively.

The results of shrinkage (left axis) and cooking yield (right axis) of the plant-based burgers are presented in Figure 5. For the shrinkage parameter, it was observed that treatments with higher mushroom levels (M_15%_ and M_20%_) showed the highest shrinkage rates, while the control showed an intermediate value. This more significant reduction in diameter in the M_15%_ and M_20%_ treatments can be attributed to the high moisture content of the mushrooms, which results in greater water release during cooking, leading to a more significant contraction of the product [18]. On the other hand, treatments with mushroom proportions of 5% and 10% (respectively, M_5%_ and M_10%_) showed lower shrinkage rates, with the M_5%_ formulation showing the most minor reduction in diameter, indicating a potential positive effect on shape retention. Lower shrinkage rates are desirable as they help maintain the product’s original shape, improving consumers’ perception of quality and freshness [31].

Regarding cooking yield, there were no significant differences between the control and the M_5%_, M_10%_, and M_15%_ treatments, all showing yields between 92% and 94%. These high values indicate that the product retained most of its initial composition, with limited weight loss during cooking. However, the formulation with a proportion of 20% mushroom (M_20%_) showed a lower cooking yield, around 89%, indicating more significant weight loss during cooking. This suggests that, due to its high moisture content, increasing the mushroom level results in greater liquid release during cooking, compromising the final yield. High cooking yield values are desirable to minimize losses and ensure the product retains a good portion of its original weight, meeting consumer expectations [21,31].

### 3.6. Sensory Analysis

**Figure 6:**
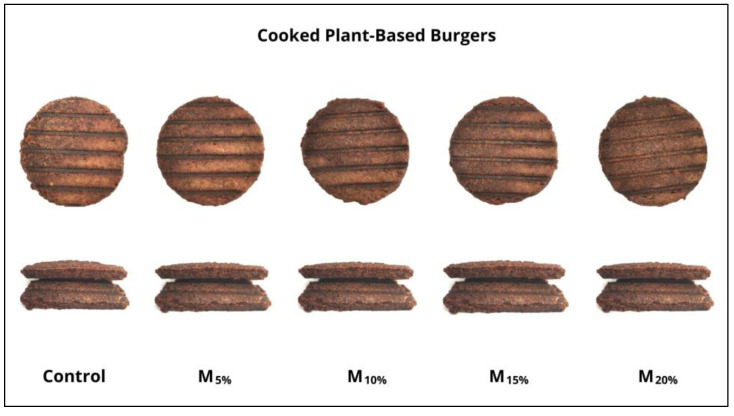
External and internal appearance of cooked plant-based burgers. Batches: control: 65.3% hydrated texturized soy protein (1:2 protein: water); M_5%_, M_10%_, M_15%_, and M_20%_: 5, 10, 15, and 20% substitution of hydrated texturized soy protein by raw mushroom, respectively.

Table 2 presents the results of the acceptance test of the plant-based burgers. No significant differences were found between the modified treatments and the control regarding aroma, flavor, texture, and overall acceptance. However, color was the attribute that stood out the most, with the M_20%_ sample showing a significantly lower value than the control. This result is consistent with the instrumental color analysis data (Figure 2), which show an increased dark tone as the proportion of mushroom increased. Formulations with mushroom proportions up to 15% maintained a color similar to the control, suggesting that replacement is feasible up to this level without compromising the visual aspect of the product. The external and internal appearance of the cooked plant-based burgers are shown in Figure 6.

**Figure 7:**
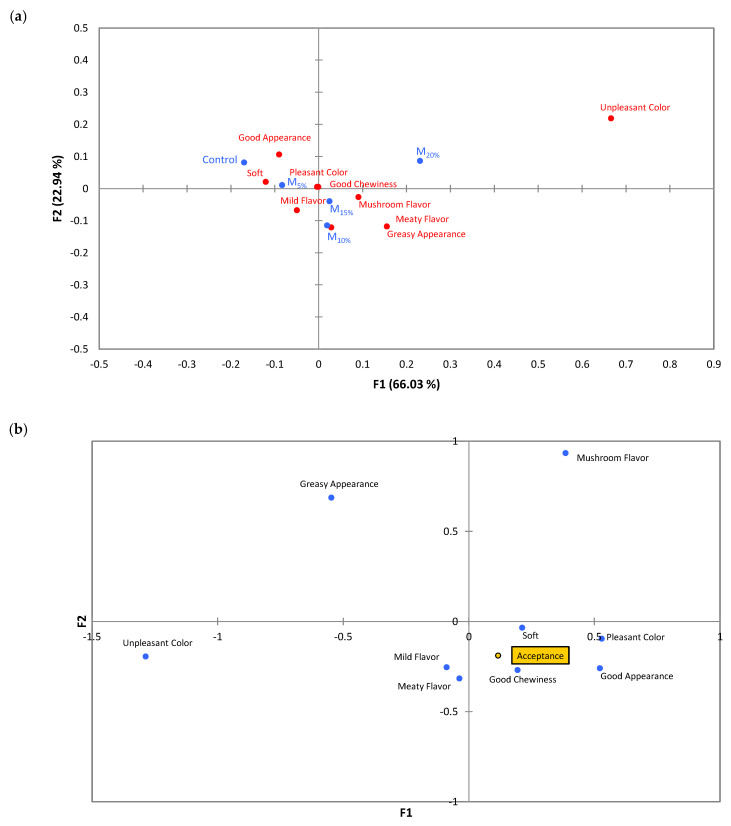
(**a**) Representation of the samples and the terms in the first and second dimensions of correspondence analysis performed on data questions on a check-all-that-apply basis (CATA). (**b**) Principal coordinate analysis applied to correlate the CATA descriptors and acceptance scores. Batches: control: 65.3% hydrated texturized soy protein (1:2 protein: water); M_5%_, M_10%_, M_15%_, and M_20%_: 5, 10, 15, and 20% substitution of hydrated texturized soy protein by raw mushroom, respectively.

The correspondence analysis of the CATA data is represented in (Figure 7a). The map explains 88.97% of the total variation in the data, distributed in two main factors: F1 (66.03%) and F2 (22.94%). The formulations were divided into four quadrants along factors F1 and F2. The control and M_5%_ formulation are grouped in the upper left quadrant and related with the attributes “soft” and “good appearance”. In the upper right quadrant, the M_20%_ treatment is characterized by “unpleasant color”, indicating a good association with the acceptance test results (Table 2). In the lower right quadrant, the M_10%_ and M_15%_ formulations are associated with the attributes “meaty flavor”, “mushroom flavor”, and “greasy appearance”. The presence of these descriptors in the CATA analysis highlights *Agaricus bisporus*’s impact on the burgers’ flavor profile, suggesting that even partial substitution of TSP introduces distinct flavor notes. Notably, these attributes are not associated with the lower (M_5%_) or higher (M_20%_) levels of mushroom inclusion, indicating a potential optimal concentration range for flavor enhancement. The attributes “pleasant color” and “good chewiness” are positioned in the central region of the map, indicating that they are balanced characteristics present in various samples, especially in the M_5%_, M_10%_, and M_15%_ formulations.

The principal coordinate analysis (Figure 7b) presents the relationship between sensory attributes and plant-based burgers’ acceptance scores. The variable “acceptance” was close to the attributes “softness”, “pleasant color”, “good chewiness”, and “good appearance”, suggesting that these aspects were the most valued by consumers. The M_5%_, M_10%_, and M_15%_ samples were positively correlated with the attributes near the acceptance point, indicating that these formulations had greater consumer preference. On the other hand, attributes such as “unpleasant color” and “greasy appearance” were distant from the acceptance variable, indicating a negative association of these factors with consumer acceptance.

## 4. Conclusions

Replacing up to 15% of texturized soy protein (TSP) with *Agaricus bisporus* mushrooms proved feasible, enhancing moisture and tenderness in plant-based burgers without compromising overall quality. This substitution maintained key sensory attributes such as color, aroma, flavor, texture, and overall acceptance, demonstrating good consumer appeal. Notably, the formulation with 5% mushroom substitution was positively associated with ‘softness,’ ‘good appearance,’ and ‘pleasant color,’ attributes that significantly contributed to product acceptance. These findings suggest that *Agaricus bisporus* mushrooms offer a promising alternative to TSP, not only for their sensory contributions but also for potential nutritional and functional benefits. Broader implications for plant-based product development include the exploration of mushrooms’ capacity to improve moisture retention and texture in low-fat formulations and their role in creating minimally processed, consumer-friendly options. Nevertheless, future studies should explore the impact of mushroom inclusion on water mobility and microstructure to clarify its effects on texture and stability further. In addition, evaluating the volatile compounds in formulations with *Agaricus bisporus* could provide a deeper understanding of how mushroom inclusion influences the flavor profile, which may help optimize sensory acceptance. Additionally, assessing the shelf-life stability of formulations with mushroom inclusion would provide valuable insights into the long-term quality of these plant-based products.

## Figures and Tables

**Table 1:** Effect of the partial replacement of hydrated texturized soy protein by *Agaricus bisporus* on the chemical composition of plant-based burgers.

(%)	Control	M_5%_	M_10%_	M_15%_	M_20%_	SEM	SIG
Moisture	53.4 ^c^	53.3 ^c^	56.0 ^b^	57.3 ^a^	57.6 ^a^	0.4	***
Fat	16.1 ^a^	16.3 ^a^	16.4 ^a^	14.9 ^a^	15.2 ^a^	0.3	n.s.
Protein	12.1 ^a^	11.3 ^ab^	11.0 ^ab^	11.3 ^ab^	9.5 ^b^	0.2	*
Ash	2.6 ^a^	2.5 ^b^	2.5 ^b^	2.5 ^b^	2.4 ^c^	0.01	***

Averages within the same line followed by the same letters did not show any significant difference (*p* > 0.05) by Tukey’s test. Batches: control: 65.3% hydrated texturized soy protein (1:2 protein: water); M_5%_, M_10%_, M_15%_, and M_20%_: 5, 10, 15, and 20% substitution of hydrated texturized soy protein by raw mushroom, respectively. SEM—standard error of the mean. SIG (Significance): *** (*p* < 0.001), * (*p* < 0.05), ns (not significant).

**Table 2:** Results of consumer study of plant-based burgers produced with partial replacement of hydrated texturized soy protein by *Agaricus bisporus* mushroom.

	Control	M_5%_	M_10%_	M_15%_	M_20%_	SEM	SIG
**Color**	7.2 ^a^	7.3 ^a^	7.1 ^ab^	7.0 ^ab^	6.5 ^b^	0.2	n.s.
**Aroma**	6.9 ^a^	6.9 ^a^	6.9 ^a^	6.6 ^a^	6.6 ^a^	0.3	*
**Flavor**	6.7 ^a^	6.7 ^a^	6.8 ^a^	6.8 ^a^	6.5 ^a^	0.3	*
**Texture**	6.6 ^a^	6.7 ^a^	6.9 ^a^	6.7 ^a^	6.7 ^a^	0.1	*
**Overall acceptance**	6.9 ^a^	6.9 ^a^	7.0 ^a^	7.0 ^a^	6.6 ^a^	0.2	*

Averages within the same line followed by the same letters did not show any significant difference (*p* > 0.05) by Tukey’s test. Batches: control: 65.3% hydrated texturized soy protein (1:2 protein: water); M_5%_, M_10%_, M_15%_, and M_20%_: 5, 10, 15, and 20% substitution of hydrated texturized soy protein by raw mushroom, respectively. SEM—standard error of the mean. SIG (significance): * (*p* < 0.05), ns (not significant).

## Data Availability

The original contributions presented in this study are included in the article. Further inquiries can be directed to the corresponding author.
